# Examining the Relationships Between Blood Cadmium, DNA Methylation Biomarker, Telomere Length, and Their Associations with Mortality in U.S. Adults

**DOI:** 10.3390/life15091467

**Published:** 2025-09-18

**Authors:** Chien-Yu Lin, Ching-Way Chen, Pei-Lun Chu

**Affiliations:** 1 School of Medicine, College of Medicine, Fu Jen Catholic University, New Taipei 242, Taiwan; 00724@km.eck.org.tw; 2 Department of Internal Medicine, En Chu Kong Hospital, New Taipei 237, Taiwan; 3 Department of Cardiology, National Taiwan University Hospital Yunlin Branch, Yunlin 640, Taiwan; y05213@ms1.ylh.gov.tw; 4 Department of Internal Medicine, Fu Jen Catholic University Hospital, Fu Jen Catholic University, New Taipei 242, Taiwan

**Keywords:** cadmium, DNA methylation, DNA methylation, mortality, National Health and Nutrition Examination Survey (NHANES), telomere length

## Abstract

Cadmium exposure has been associated with shortened telomeres, alterations in DNA methylation patterns, and increased mortality. However, the role of DNA methylation in mediating the relationship between cadmium and telomere dynamics is still unclear. Additionally, it is unknow how telomere dynamics and DNA methylation alterations may affect the association between cadmium exposure and mortality outcomes. We utilized data from 8716 National Health and Nutrition Examination Survey (NHANES) participants aged 18 and above, collected between 1999 and 2002, and linked these to mortality outcomes from the National Center for Health Statistics (NCHS) through 2019. In the final model, ln-blood cadmium was significantly and inversely associated with ln-T/S ratio (β = −0.043, 95% CI: −0.059 to −0.027, *p* < 0.001), while ln-Horvath DNAmTL was strongly and positively associated with ln-T/S ratio (β = 1.782, 95% CI: 1.467 to 2.097, *p* < 0.001). Moreover, ln-blood cadmium also showed a significant inverse association with ln-Horvath DNAmTL (β = −0.010, 95% CI: −0.014 to −0.006, *p* < 0.001). Structural equation modeling showed that the association between cadmium and T/S ratio was mediated by Horvath DNAmTL, with a total effect of −0.044, a direct effect of −0.027, and an indirect effect of −0.017. Furthermore, stratified analyses revealed that a 1-unit increase in ln-blood cadmium was associated with higher all-cause mortality, with hazard ratios (HR) of 1.47 for participants with T/S ratio below the median and 1.41 for those above. Similar patterns were observed for cardiovascular (HR = 1.68 vs. 1.30) and cancer mortality (HR = 1.75 vs. 1.42). For Horvath DNAmTL, the association was significant only for all-cause mortality (HR = 1.36 vs. 1.31). However, no significant interactions were detected. In conclusion, our findings suggest that Horvath DNAmTL is associated with the relationship between cadmium and telomere length, suggesting a potential DNA methylation pathway that warrants further longitudinal investigation. Individuals with lower T/S ratios or Horvath DNAmTL appear to be more susceptible to cadmium-related mortality. Further research is necessary to confirm these results.

## 1. Introduction

Cadmium is a toxic heavy metal commonly encountered in the environment through cigarette smoke, contaminated food, and industrial emissions [1]. Its long biological half-life allows it to persist in these organs for decades. Once in systemic circulation, cadmium binds to proteins like metallothionein and accumulates primarily in the liver and kidneys, with minimal excretion [2]. Cadmium exposure induces oxidative stress, damaging lipids, proteins, and DNA, leading to adverse effects on multiple organ systems [3]. Due to its widespread presence and well-documented associations with chronic conditions such as kidney disease, cardiovascular disease (CVD), and cancer, numerous strategies have been implemented to minimize cadmium exposure [4]. Nevertheless, emerging research continues to demonstrate that even low-level cadmium exposure poses substantial health hazards and is linked to elevated mortality risk [5,6,7].

Telomeres are protective structures at the ends of chromosomes that maintain genomic integrity. They shorten with each cell division, and when they become critically short they trigger cellular senescence or apoptosis [8]. Shortened telomeres are associated with age-related conditions such as CVD and cancer, and are linked to an increased risk of mortality, positioning telomere length as a potential indicator of lifespan and overall health [9]. Beyond natural aging, cadmium exposure accelerates telomere shortening by inducing reactive oxygen species (ROS), which cause oxidative damage to DNA [10]. Epidemiological studies indicate that higher cadmium exposure is associated with shorter telomeres, suggesting a potential mechanism through which cadmium may impact biological aging and related health risks [11,12].

Epigenetic changes regulate gene activity without altering the DNA sequence [13]. Among these, DNA methylation—characterized by the attachment of methyl groups to the C5 position of cytosine within CpG sites—is both inheritable and environmentally responsive [14]. Cadmium has been shown to interfere with methylation patterns in both experimental and animal studies [15,16,17]. Population-based research also links cadmium exposure to methylation changes, suggesting a potential pathway through which cadmium contributes to negative health effects [18,19,20]. DNA methylation influences telomere length by modulating the expression of telomere maintenance genes like telomerase and shelterin [21,22]. Changes in DNA methylation, particularly in subtelomeric regions, can either accelerate telomere shortening or stabilize them [23]. Among the various DNA methylation biomarkers, Horvath DNA methylation-predicted telomere length (Horvath DNAmTL) is a DNA methylation estimator derived from ~140 CpG sites that not only predicts leukocyte telomere length but also captures long-term biological aging signatures in the methylome, reflecting cumulative proliferative history and epigenetic aging processes. While correlated with telomere biology, it provides related yet distinct information and may offer complementary insights into cellular aging and disease risk [24]. A recent exposome-wide study found that higher cadmium exposure was significantly associated with shorter Horvath DNAmTL and accelerated aging measured by clocks like GrimAge [25].

Although previous studies have shown that cadmium exposure is associated with chronic diseases, shorter leukocyte telomere length, and reduced Horvath DNAmTL, no study has yet investigated these relationships concurrently in a nationally representative cohort. In particular, it remains unclear whether DNA methylation telomere predictors, such as Horvath DNAmTL, mediate the impact of cadmium on telomere shortening and whether these biomarkers jointly predict long-term mortality outcomes. Leveraging NHANES 1999–2002 with mortality follow-up through 2019, our study is the first to comprehensively evaluate cadmium exposure in relation to both measured telomere length and Horvath DNAmTL, to explore mediation pathways, and to assess their combined relevance for mortality risk in the U.S. adult population.

## 2. Materials and Methods

### 2.1. Study Population

**Figure 1:**
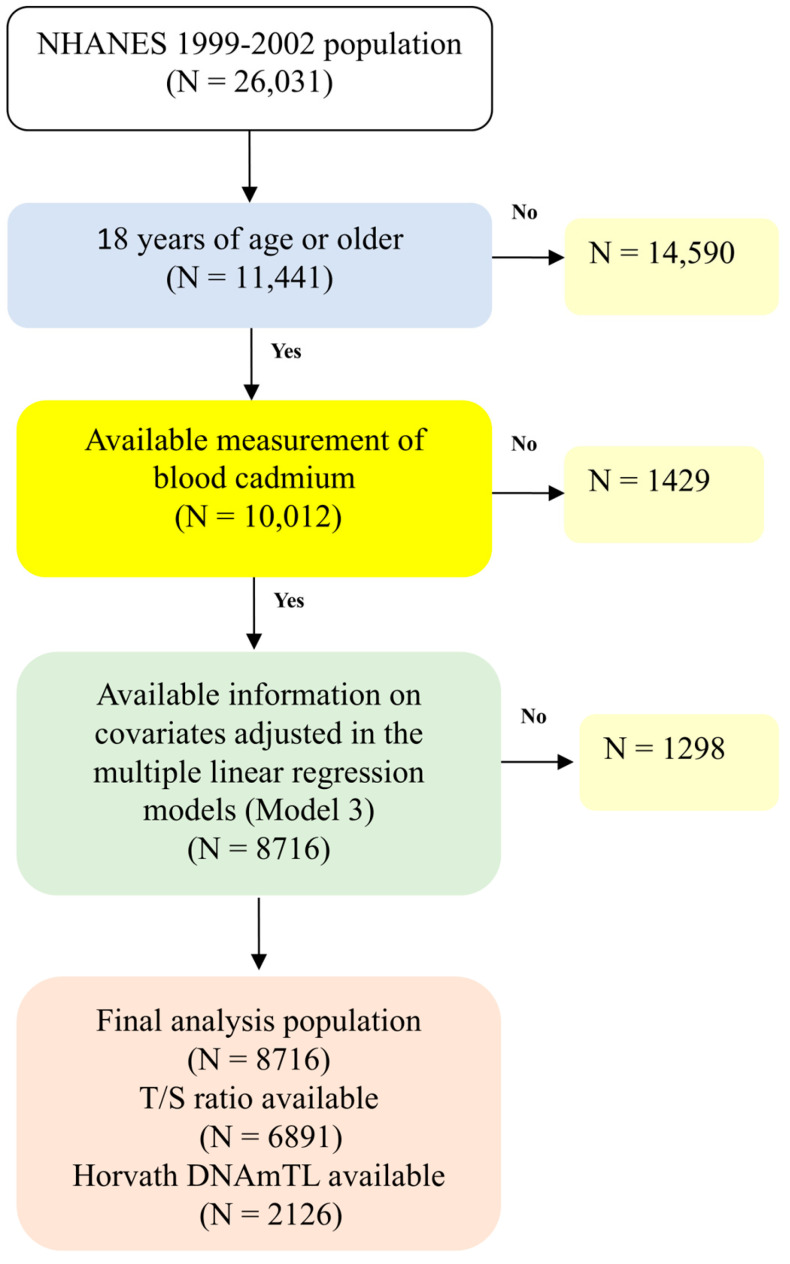
Flow chart algorithm.

NHANES is a biannual survey designed to provide nationally representative health and nutrition data on the non-institutionalized U.S. population through a complex, multistage probability sampling design. Methodological details and consent procedures are available on the NHANES website [26]. All biological measurements were conducted by CDC/National Center for Health Statistics (NCHS) laboratories under the NHANES protocol, and our study represents a reanalysis of these publicly available data. This study analyzed data from the 1999–2002 cycles, which initially included 26,031 individuals. After restricting to adults aged ≥ 18 years and those with available blood cadmium data, 10,012 participants remained. We further excluded individuals with missing covariates required for regression modeling (Model 3 covariates), leaving 8716 participants in the final analytic sample. For outcomes involving telomere length (T/S ratio), analyses were restricted to 6891 participants aged ≥ 20 years with available data. For outcomes involving Horvath DNAmTL, analyses were inherently restricted to 2126 participants aged ≥ 50 years, as this biomarker was only assayed in that age group. The participant selection flow is shown in Figure 1.

### 2.2. Measurement of Blood Cadmium

In the 1999–2002 NHANES cycles, blood cadmium was measured in participants aged 1 year and older; this analysis focused on adults aged 18 and above. Cadmium levels were determined by the National Center for Environmental Health (Atlanta, GA, USA) using a PerkinElmer SIMAA 6000 atomic absorption spectrometer (PerkinElmer Inc., Waltham, MA, USA) with Zeeman background correction. The method detects cadmium absorption at 228.8 nm, with specimens diluted in a solution of nitric acid, Triton X-100, and ammonium phosphate. The limit of detection (LOD) was 0.3 µg/L, and values below the LOD were imputed as LOD divided by √2. Full methodological details are available on the NHANES website [27].

### 2.3. Measurement of Telomere Length

In NHANES 1999–2002, participants aged 20 and older who provided blood for DNA analysis had genomic DNA extracted from whole blood using the Puregene (D-50K) kit (D-50K; Qiagen, Germantown, MD, USA) and stored at −80 °C. Telomere length was assessed via quantitative PCR, which calculates the T/S ratio. Quality control was maintained by the CDC through the inclusion of five 96-well plates (5% of total samples), requiring at least 95% agreement between duplicates and an inter-assay coefficient of variation below 6.5%. Full methodological details are available on the NHANES website [28].

### 2.4. Measurement of Horvath DNAmTL

In NHANES, this study included adults aged 50 and older from the 1999–2002 cycles who had available DNA samples. The cohort consisted of all eligible participants from minority racial/ethnic groups and a random subset of non-Hispanic White individuals. DNA methylation profiling was performed on whole blood by CDC/NCHS laboratories using the Illumina Infinium MethylationEPIC BeadChip (Illumina Inc., San Diego, CA, USA), with standardized preprocessing, normalization, and batch correction procedures. As DNAmTL was only available for participants aged ≥ 50 years, all analyses involving this biomarker are inherently restricted to this age group. Detailed methodological information is available on the NHANES website [29].

### 2.5. Covariates

Sociodemographic data—including age, sex, and race/ethnicity—were obtained from the NHANES database. Smoking status was classified as current smoker, secondhand smoke exposure (ETS), or non-smoker, based on questionnaire responses and serum cotinine levels [30]. Alcohol intake was determined by self-reported consumption of at least 12 alcoholic drinks in the past year. Body mass index (BMI) was calculated as weight (kg) divided by height squared (m^2^). Hypertension was defined by either self-reported use of antihypertensive medications or an average blood pressure ≥ 140/90 mmHg. Diabetes was identified by a fasting glucose ≥ 126 mg/dL, HbA1c ≥ 6.5%, or use of anti-diabetic drugs. Hypercholesterolemia was defined as a fasting LDL ≥ 130 mg/dL or current cholesterol-lowering treatment. Chronic kidney disease (CKD) was determined by an estimated glomerular filtration rate (eGFR) < 60 mL/min/1.73 m^2^ [31]. Cardiovascular disease (CVD) history included self-reported diagnoses of heart failure, coronary heart disease, angina, myocardial infarction, or stroke. History of cancer was determined from self-reported questionnaire data in NHANES, based on the question “Have you ever been told by a doctor or other health professional that you had cancer or a malignancy of any kind?” As such, this variable encompassed all cancer types combined rather than distinguishing specific cancer sites [32].

### 2.6. Outcomes

The NCHS linked NHANES 1999–2002 data to the National Death Index, providing mortality follow-up through 31 December 2019. Follow-up time was calculated from the baseline examination date until death or censoring at 31 December 2019, whichever came first. Outcomes were limited to mortality and classified as all-cause, cardiovascular-related, or cancer-related deaths. Cardiovascular mortality included deaths due to heart disease or cerebrovascular events. Additional methodological details are available on the NCHS website [33].

### 2.7. Statistics

The natural logarithm (ln) of blood cadmium, T/S ratio, and Horvath DNAmTL concentrations was applied to calculate the exponential mean, allowing for the determination of the geometric mean and standard error across different subgroups. Statistical analyses were performed using two-tailed Student’s *t*-tests and one-way analysis of variance. To account for the NHANES design, we applied examination full sample 4-year mobile examination center examination weight (WTMEC4YR) provided by NCHS, which adjust for differential probabilities of selection, oversampling of specific subgroups, and nonresponse. All analyses incorporated the sample weights, along with strata and primary sampling units, to yield unbiased, nationally representative estimates. Multiple linear regression and survival analyses were conducted using complex survey procedures that accounted for NHANES’s multistage, stratified, probability cluster design [34]. We conducted multiple linear regression analyses using complex sampling techniques to explore the relationship between blood cadmium levels, the T/S ratio, and Horvath DNAmTL. Model 1 included core demographic and lifestyle variables such as age, sex, ethnicity, family poverty income ratio, smoking status, alcohol consumption, and BMI, which are well-established determinants of both cadmium exposure and telomere length. Model 2 built upon this by adding clinical variables for hypertension, hypercholesterolemia, diabetes, and chronic kidney disease, conditions linked to oxidative stress and telomere attrition. Model 3 further incorporated a history of cardiovascular disease and cancer to account for the impact of major chronic illnesses that could independently affect telomere shortening and mortality risk. These covariates were selected because they are established determinants of both cadmium exposure and telomere biology. For instance, cadmium burden varies by age, sex, smoking, and socioeconomic status, while telomere length and DNAmTL are strongly influenced by age, sex, obesity, and chronic conditions such as hypertension, diabetes, and CKD. Including these covariates helps to account for potential confounding and better isolate the associations of interest. Only results that remained statistically significant across all three models were interpreted as robust [35,36]. We additionally performed survey-weighted linear regression analyses of T/S ratio and Horvath DNAmTL across quartiles of serum cadmium and Horvath DNAmTL. All models were adjusted for Model 3 covariates. Pairwise comparisons with the lowest quartile were assessed using Sidak-adjusted contrasts, and P for trend was obtained by modeling the quartile medians as a continuous variable in the Wald F test. We assessed the odds ratio (OR) of chronic diseases associated with a one-unit increase in ln-blood cadmium, ln-T/S ratio, and ln-Horvath DNAmTL using logistic regression analysis for complex samples, adjusting for the covariates in Model 1.

In this study, Structural Equation Modeling (SEM) was used to explore the relationships between blood cadmium, with Horvath DNAmTL as a mediator and the T/S ratio as the outcome. It was hypothesized that blood cadmium could influence the T/S ratio either directly or indirectly through Horvath DNAmTL. The model adjusted for Model 3 covariates and was estimated using generalized least squares with the CALIS procedure. Model fit was assessed using the goodness of fit index (GFI), normed fit index (NFI), and root mean square residual (RMR). A GFI and NFI greater than 0.9 and an RMR below 0.05 were considered indicators of a good model fit. The estimates of the parameters and the overall model fit were reported.

To understand the relationship between blood cadmium, telomere metrics, and mortality, we assessed hazard ratios (HR) for all-cause, cardiovascular, and cancer-related mortality associated with a one-unit increase in ln-blood cadmium, ln-T/S ratio, and ln-Horvath DNAmTL. This analysis was conducted using a weighted Cox regression model and adjusted for covariates in Model 3. To reinforce our findings, we conducted a sensitivity analysis excluding individuals with a history of CVD and cancer, as well as excluding active smokers and those exposed to ETS. Additionally, we assessed HRs for mortality linked to a unit increase in ln-blood cadmium across different subgroups defined by T/S ratio and Horvath DNAmTL. Furthermore, we conducted analyses to test interactions between blood cadmium and DNA methylation biomarkers by incorporating cross-product terms into Cox regression models. Because blood cadmium, T/S ratio, and Horvath DNAmTL exhibited right-skewed distributions, ln transformation was applied to improve normality and model fit, consistent with prior NHANES-based studies of metals and telomere length [7,37]. All descriptive analyses, regression models, and survival analyses were conducted using IBM SPSS Statistics, version 30.0 (IBM Corp., Armonk, NY, USA), while SEM analyses were performed using the CALIS procedure in SAS, version 9.4 (SAS Institute Inc., Cary, NC, USA). A two-sided *p* < 0.05 was considered statistically significant.

## 3. Results

The studied participants had a mean age (SD) of 45.50 (19.77) years, ranging from 18 to 85 years old. Approximately 75.9% of individuals showed detectable levels of blood cadmium. Over a median follow-up of 219.0 months, data for 4 participants were missing. A total of 2153 deaths were recorded, including 670 cardiovascular-related and 462 cancer-related deaths. Group comparisons of baseline characteristics were assessed using *t*-tests or ANOVA, while regression and survival analyses accounted for the NHANES complex sampling design, as described in the Methods section. Table 1 summarizes the demographic characteristics and key biomarkers, including blood cadmium levels, T/S ratio, and Horvath DNAmTL. Significant findings show that older individuals and those with hypertension, chronic kidney disease, a history of CVD, or a history of cancer have higher blood cadmium levels and lower T/S ratios and Horvath DNAmTL. Women and individuals with a family poverty income ratio between 1–3 tend to have higher T/S ratios and Horvath DNAmTL. Higher blood cadmium levels are observed in individuals of other ethnicities, lower income groups, lower BMI, active smokers, those who consume ≥ 12 drinks per year, and individuals with hypercholesterolemia. Lower T/S ratios are found among Mexican Americans, individuals with higher BMI, non-smokers, and those with diabetes mellitus and hypercholesterolemia. Non-Hispanic whites and individuals with lower BMI tend to have lower Horvath DNAmTL.

In Supplemental Table S1, complex samples of logistic regression analysis was performed to examine the OR for chronic diseases with one-unit increases in ln-blood cadmium, ln-T/S ratio, and ln-Horvath DNAmTL, adjusting for Model 1. For ln-blood cadmium, a significant positive association was found with chronic kidney disease (OR: 1.401, 95% CI: 1.048–1.872, *p* = 0.024), history of CVD (OR: 1.291, 95% CI: 1.039–1.604, *p* = 0.023), and history of cancer (OR: 1.341, 95% CI: 1.076–1.671, *p* = 0.011). Ln-T/S ratio showed a significant positive association with hypertension (OR: 1.458, 95% CI: 1.073–1.981, *p* = 0.018), but a negative association with history of CVD (OR: 0.524, 95% CI: 0.282–0.972, *p* = 0.041). Ln-Horvath DNAmTL was negatively associated with history of CVD (OR: 0.011, 95% CI: 0.000–0.407, *p* = 0.016), while other associations were not significant.

Table 2 presents adjusted regression coefficients for differences in ln-T/S ratio and ln-Horvath DNAmTL relative to a one-unit increase in ln-blood cadmium and ln-Horvath DNAmTL, with results weighted for sampling strategy. In all models, ln-blood cadmium was negatively associated with ln-T/S ratio across 6891 participants representing a population size of 150,739,685 (β = −0.043, 95% CI: −0.059 to −0.027, *p* < 0.001 in Model 3). Similarly, ln-Horvath DNAmTL was positively associated with ln-T/S ratio in all models (β = 1.785, 95% CI: 1.469 to 2.101, *p* < 0.001 in Model 3). For ln-Horvath DNAmTL, ln-blood cadmium was negatively associated across 2126 participants representing a population size of 32,034,886 (β = −0.010, 95% CI: −0.014 to −0.006, *p* < 0.001 in Model 3).

**Figure 2:**
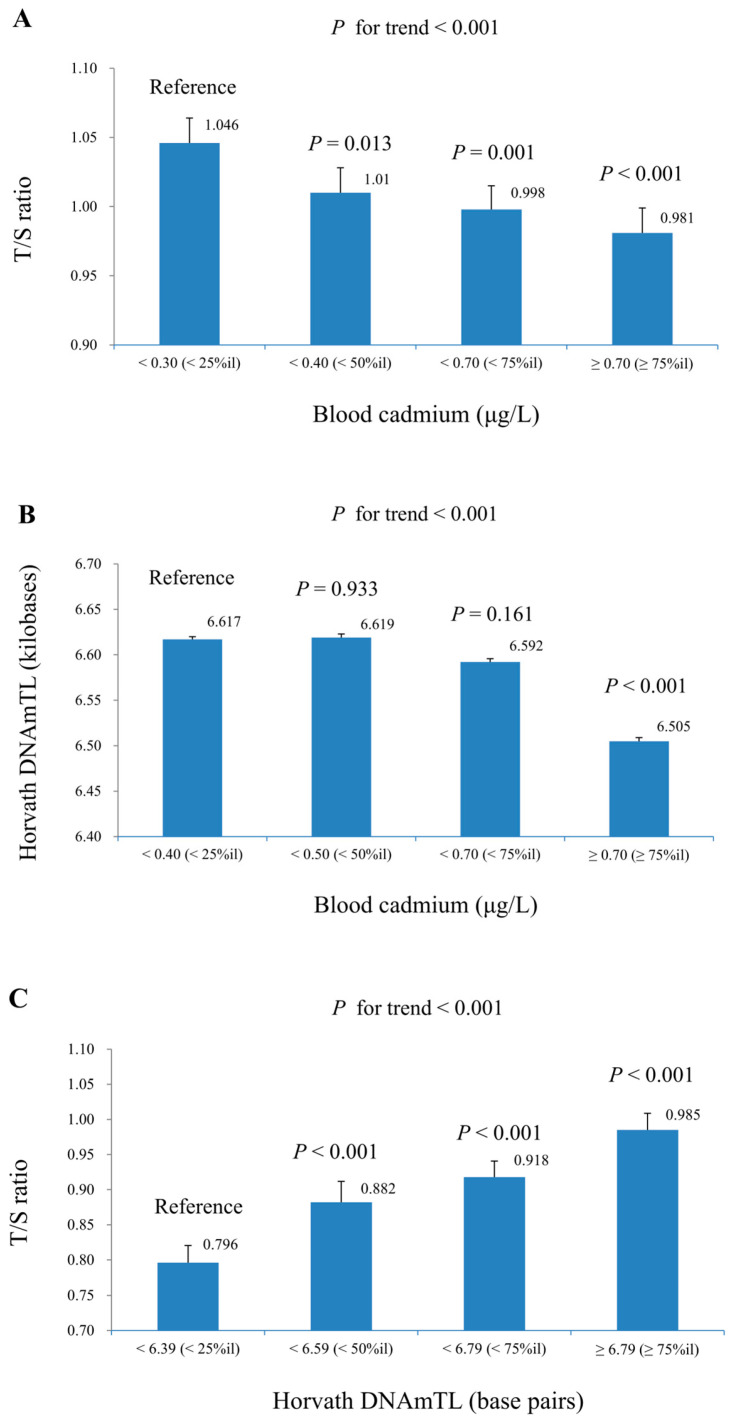
Geometric mean (±standard error) of dependent variables across quartiles of the independent variables in survey-weighted multiple linear regression models adjusted for Model 3 covariates. Results account for NHANES complex sampling design. (**A**) T/S ratio by quartiles of serum cadmium. (**B**) Horvath DNAmTL by quartiles of serum cadmium. (**C**) T/S ratio by quartiles of Horvath DNAmTL.

Figure 2 summarizes the geometric means of Horvath DNAmTL and T/S ratio across quartiles of blood cadmium and Horvath DNAmTL, based on complex sample multiple linear regression models. The analysis shows a significant decrease in both T/S ratio and Horvath DNAmTL as blood cadmium quartiles increase, while T/S ratio significantly increases across higher quartiles of Horvath DNAmTL. All trend *p*-values were less than 0.001, indicating strong statistical significance. Table 3 presents the linear regression results for ln-T/S ratio and ln-Horvath DNAmTL per unit increase in ln-blood cadmium across various subpopulations. All outcomes showed a negative association with blood cadmium. Significant interactions were only observed for age (*p* = 0.035) in the association between blood cadmium and T/S ratio, while other subgroup differences were not statistically significant. Given the number of subgroup comparisons performed, these results should be considered exploratory, and no formal correction for multiple testing was applied.

**Figure 3:**
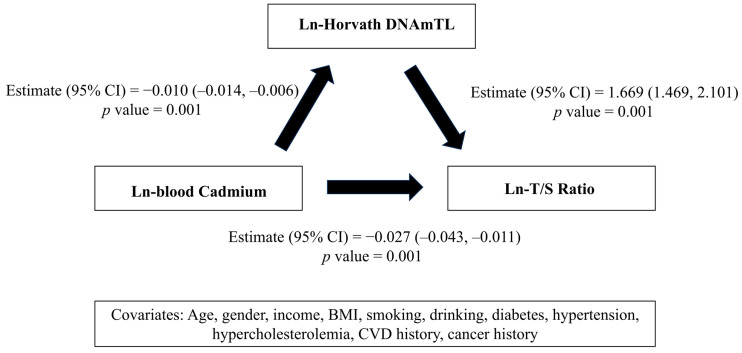
The relationship between blood cadmium, T/S ratio, and Horvath DNAmTL in the SEM.

Figure 3 presents the SEM results examining the relationships among blood cadmium, T/S ratio, and Horvath DNAmTL. SEM analysis identified significant inverse associations between blood cadmium and both Horvath DNAmTL (Estimate = −0.010, *p* = 0.001) and T/S ratio (Estimate = −0.027, *p* = 0.001). A strong positive link was also observed between Horvath DNAmTL and T/S ratio (Estimate = 1.669, *p* = 0.001). Model fit was adequate according to established SEM criteria [38], with goodness-of-fit index (GFI) of 0.892, normed fit index (NFI) of 0.910, and root mean square error (RMS) of 0.050. The total effect of blood cadmium on T/S ratio was −0.044, including a direct effect of −0.027 and an indirect effect of −0.017 mediated through Horvath DNAmTL.

Table 4 presents the HRs for all-cause, cardiovascular, and cancer-related mortality associated with ln-blood cadmium, ln-T/S ratio, and ln-Horvath DNAmTL in a composite model using weighted Cox regression. ln-blood cadmium was significantly associated with increased risk of all three mortality outcomes. The ln-T/S ratio was identified as a predictor for both all-cause and cardiovascular mortality. Additionally, ln-Horvath DNAmTL was found to be a robust predictor for all-cause mortality as well as cancer-related mortality. In Supplemental Table S2, a sensitivity analysis was performed by excluding individuals with a history of CVD and cancer. The associations between the three biomarkers and mortality outcomes remained consistent, with the only notable difference being that the association between the T/S ratio and cardiovascular mortality became insignificant. In Supplemental Table S3, a sensitivity analysis was conducted by excluding individuals who were active smokers or exposed to ETS. The associations between the three biomarkers and mortality outcomes remained consistent. The notable differences were that the association between the T/S ratio and cardiovascular mortality became insignificant, while the association between the T/S ratio and cancer-related mortality became significant.

Table 5 presents hazard ratios (HRs) for all-cause, cardiovascular, and cancer-related mortality associated with a 1-unit increase in ln-blood cadmium, stratified by subgroups of T/S ratio and Horvath DNAmTL. Across all T/S ratio strata, blood cadmium remained significantly associated with each mortality outcome. Specifically, a 1-unit rise in ln-blood cadmium increased all-cause mortality by 47% in the short-telomere group (T/S < median; HR = 1.47) and 41% in the long-telomere group (HR = 1.41). This difference was more pronounced for cardiovascular mortality (HR = 1.68 vs. 1.30) and cancer-related mortality (HR = 1.75 vs. 1.42), suggesting that shorter T/S ratios may amplify the adverse effects of cadmium exposure. In contrast, for Horvath DNAmTL, the association with cadmium was significant only for all-cause mortality, with HRs of 1.36 and 1.31 for the < median and ≥ median subgroups, respectively, and was not significant for cause-specific mortality. However, no significant interactions were observed between blood cadmium and either T/S ratio or Horvath DNAmTL for any of the mortality outcomes, indicating a lack of synergistic modification.

## 4. Discussion

In a nationally representative sample of U.S. adults, we identified a negative association between blood cadmium and telomere length, with this relationship partially mediated by a DNA methylation biomarker predictive of telomere length in cross-sectional analysis. Additionally, blood cadmium was significantly associated with all mortality outcomes, while both telomere length and Horvath DNAmTL showed predictive effects across several mortality outcomes. Although no significant interactions were found, individuals below the 50th percentile for T/S ratio and Horvath DNAmTL had higher HRs for cadmium-related mortality. These findings suggest that those with lower T/S ratios or DNAmTL may be more vulnerable to mortality associated with cadmium exposure. This study is the first to investigate the interconnections between blood cadmium levels, a DNA methylation-based predictor of telomere length, measured telomere length, and their associations with mortality risk. Our findings point to a possible mechanistic pathway through which cadmium exposure may drive DNA methylation alterations, disrupt telomere maintenance, and contribute to increased mortality. These results enhance our understanding of how environmental contaminants like cadmium may accelerate biological aging and impact long-term health outcomes.

In the current study, we observed that men exhibited shorter telomere lengths and lower DNA methylation levels. These findings may reflect gender differences in DNA methylation modifications and telomere attrition and are consistent with previous studies [39]. Age-related trends showed increasing blood cadmium levels and decreasing telomere length and DNA methylation with age, highlighting potential age-related cadmium accumulation and accelerated aging. Ethnic differences were observed, reflecting variability in exposure and biological responses. Non-Hispanic black individuals had longer telomeres compared to non-Hispanic whites, consistent with previous research [40]. Another NHANES study found this difference was most pronounced among those with lower socioeconomic status, suggesting that social and environmental factors, rather than genetics alone, may contribute to longer telomeres in this population [40]. Moreover, lifestyle factors, such as smoking, had a marked effect on cadmium levels and telomere metrics. Current smokers had the highest blood cadmium levels, while non-smokers had lower levels. Due to the extensive use of chemical fertilizers in tobacco cultivation, cigarettes are often contaminated with cadmium [41]. Telomere lengths followed a similar trend, with smokers exhibiting slightly longer T/S ratios compared to non-smokers (1.026 vs. 0.979). This unexpected finding warrants further investigation and may be influenced by survivor bias as well as unmeasured confounding factors. Health conditions such as hypertension, diabetes, chronic kidney disease, and a history of CVD or cancer were associated with higher cadmium levels, shorter telomeres, and lower Horvath DNAmTL. This underscores the relationship between chronic diseases, cadmium exposure, and biological aging in a cross-sectional context. These findings underscore the need to consider demographic, socioeconomic, and health-related factors when evaluating the effects of cadmium on aging and health.

### 4.1. Cadmium and Telomere Length

Cadmium triggers oxidative stress and promotes the production of ROS, leading to cellular damage, notably DNA. This process accelerates telomere shortening, as telomeres are particularly vulnerable to oxidative damage [3]. The association between cadmium exposure and telomere shortening observed in our study has also been reported in previous research using the 1999–2002 NHANES database [37]. Additionally, similar findings have been reported in birth cohort studies from Myanmar and China [11,42], among pregnant women in high-exposure areas of China [43], and in adolescents from Nepal [12]. However, not all studies have reached the same conclusion. For example, a study among university students in Japan did not observe this association [44]. These findings suggest that cadmium exposure may be linked to telomere shortening, a potential marker of biological aging, as observed in various populations. However, the inconsistency across studies, highlights that the effect of cadmium on telomeres may vary by population or other factors.

### 4.2. Cadmium and Horvath DNAmTL

We also observed that blood cadmium level was negatively associated with Horvath DNAmTL. Horvath DNAmTL is a DNA methylation biomarker that assesses DNA methylation patterns across CpG sites linked to biological aging, including telomere dynamics, rather than focusing solely on telomere-specific regions [24]. The result indicated that cadmium exposure may alter DNA methylation regulation. Experimental evidence indicates that cadmium disrupts DNA-methylation dynamics. Chronic exposure drives DNA methyltransferase-mediated hypermethylation of the p16INK4a promoter, removing a critical cell-cycle brake and promoting unchecked lymphocyte proliferation [15]. Prenatal exposure likewise increases CpG methylation of the fetal hepatic glucocorticoid-receptor locus, down-regulating its transcription and illustrating in vivo DNA methylation re-programming [16]. Additional data show that cadmium also hampers active demethylation, intensifies oxidative stress, and hastens telomere attrition—a triad that destabilizes the genome and elevates carcinogenic risk [17]. Several epidemiological studies have also identified a link between cadmium exposure and DNA methylation changes, including studies of adults in high-exposure areas of Thailand [45], a U.S. birth cohort [18], and the Strong Heart Study [46]. Our findings are consistent with previous studies and are the first to demonstrate this association in a U.S. nationally representative cohort.

### 4.3. The Association Between Blood Cadmium, Horvath DNAmTL, and T/S Ratio

Previous studies have shown that DNA methylation influences telomere length by regulating the expression of telomere maintenance genes, such as telomerase and shelterin [21,22]. Methylation changes, particularly in subtelomeric regions, can either accelerate telomere shortening or stabilize telomeres [23]. Horvath DNAmTL, as noted earlier, predicts telomere length based on CpG sites associated with telomere dynamics, rather than being confined to telomere-specific regions [24]. Although cadmium exposure is associated with both DNA methylation and telomere length, its role in mediating the relationship between cadmium and telomere shortening has not been previously reported. Our SEM results suggest that Horvath DNAmTL plays a substantial role in mediating this association. The total effect of ln-blood cadmium on the ln-T/S ratio is −0.044, with a direct effect of −0.027 and a notable indirect effect of −0.017 through ln- Horvath DNAmTL. This indicates that a significant portion of the negative association between cadmium and telomere length may be mediated by its impact on DNA methylation. Our findings are the first to highlight a potential DNA methylation pathway through which cadmium may affect telomere length, suggesting a potential DNA methylation pathway that warrants further longitudinal investigation.

### 4.4. Blood Cadmium, Telomere Length, DNA Methylation, and Mortality Outcomes

We observed significant associations between elevated blood cadmium levels and an increased risk of all three mortality outcomes. These findings are consistent with prior studies utilizing the 1999–2004 and 1999–2014 NHANES databases [6,7], as well as other cohort studies that have linked cadmium exposure to increased mortality risk [5]. Notably, our analysis also demonstrated that a lower T/S ratio was associated with a higher risk of mortality. This link between telomere length and mortality has been previously reported in NHANES [47] and UK Biobank studies [48]. Our findings add to the growing body of evidence highlighting the detrimental effects of cadmium exposure and telomere shortening on long-term health and lifespan. Additionally, we observed that Horvath DNAmTL was associated with both all-cause and cancer-related mortality. Previous studies have demonstrated that DNA methylation age in blood predicts mortality in various populations, including a Scottish birth cohort [49], the elderly population in Germany [50], and the Finnish Twin Study [51]. Previous studies have shown that DNA methylation is more accurate than telomere length for age estimation [52,53]. Our study is the first to report that Horvath DNAmTL serves as a predictor of mortality in a representative sample of the general middle-aged U.S. population. In terms of prognostic value, our findings suggest that measured T/S ratio and Horvath DNAmTL each capture distinct aspects of biological aging. The T/S ratio showed stronger associations with cardiovascular mortality, whereas Horvath DNAmTL was more predictive of cancer-related and all-cause mortality. These differences likely reflect their biological underpinnings. Rather than one marker being uniformly superior, both appear to provide complementary insights into mortality risk.

### 4.5. Modification Effects of T/S Ratio and Horvath DNAmTL on Cadmium Related Mortality

Since both DNA methylation modifications and telomere length are recognized as biomarkers of biological aging and health, it is plausible that they mediate the effects of cadmium on health outcomes. Several studies support this: cadmium-related methylation changes have been linked to lower birth weight in Bangladesh [54] and subclinical atherosclerosis in young populations in Taiwan [19]. Additionally, telomere length has been found to mediate the relationship between cadmium exposure and cognitive function in older U.S. adults [55]. A similar mediating role of telomere length has also been observed in cadmium-exposed Chinese populations with hypertension [56]. In this study, individuals with T/S ratios and Horvath DNAmTL below the 50th percentile exhibited higher HRs for cadmium-related mortality. However, no significant interactions were observed between the T/S ratio or Horvath DNAmTL in the relationship between cadmium exposure and mortality outcomes. This suggests a greater susceptibility to the harmful effects of cadmium exposure among these groups. Shorter telomeres and lower Horvath DNAmTL may reflect cellular aging and reduced cellular function, impairing the body’s ability to cope with environmental stressors like cadmium, potentially leading to increased mortality. However, the lack of significant interactions between T/S ratio and Horvath DNAmTL in relation to cadmium exposure suggests that while these markers independently predict higher mortality risk, they do not interact synergistically. These results highlight the vulnerability of individuals with lower T/S ratios or DNAmTL to cadmium-related mortality, emphasizing the need for targeted public health initiatives.

The public health implications of these findings are significant, as they suggest that cadmium exposure may contribute to adverse health outcomes by accelerating biological aging processes, as evidenced by shortened telomeres and DNA methylation alterations. Since telomere shortening and disrupted DNA methylation are linked to increased risks of mortality, reducing cadmium exposure in the population could potentially mitigate these health risks. Public health interventions aimed at minimizing cadmium exposure, particularly in high-risk populations, could play a crucial role in preventing premature aging, reducing disease burden, and lowering mortality rates.

It’s important to recognize both the strengths and limitations of this study. This study has several strengths, including the use of a large, nationally representative sample from NHANES, which enhances the generalizability of the findings. The integration of comprehensive mortality data linked to the NCHS allowed for an in-depth analysis of all-cause, cardiovascular, and cancer-related mortality over a long follow-up period. Additionally, the inclusion of both telomere length and a DNA methylation biomarker (Horvath DNAmTL) provided a more holistic view of the biological pathways involved. Advanced statistical methods, such as SEM, was employed to assess both direct and indirect effects, further strengthening the analysis. A key limitation is that both the T/S ratio and Horvath DNAmTL were measured only once at baseline, precluding assessment of longitudinal telomere dynamics. Future studies are needed to confirm whether Horvath DNAmTL mediates cadmium’s effect on telomere length. Another limitation is that multiple subgroup comparisons were conducted without adjustment for multiple testing, which increases the likelihood of type I error; thus, subgroup findings should be interpreted with caution. Additionally, residual confounding from unmeasured variables may have influenced the observed associations. Lastly, the attenuation of associations in the composite analysis suggests that the interplay between cadmium exposure, telomere dynamics, and DNA methylation modifications is complex and requires further research. Future longitudinal and mechanistic studies are warranted to clarify the causal pathways linking cadmium exposure, DNA methylation aging processes such as Horvath DNAmTL, and telomere biology, thereby enhancing the translational relevance of these findings for public health interventions.

## 5. Conclusions

Utilizing a nationally representative U.S. sample, our findings indicate that higher blood cadmium levels are significantly linked to shorter telomeres and lower Horvath DNAmTL, with the latter partially mediating the impact of cadmium on telomere length. Furthermore, our results suggest that individuals with lower T/S ratios or DNAmTL may be more susceptible to mortality related to cadmium exposure. These results highlight the complex interplay between cadmium exposure, telomere dynamics, DNA methylation modifications, and mortality, suggesting a need for further research to clarify the underlying mechanisms. Public health interventions to reduce cadmium exposure, especially among high-risk populations, could be pivotal in mitigating premature aging, decreasing disease burden, and lowering mortality rates.

## Figures and Tables

**Table 1:** Basic demographics of the sample subjects, including geometric means (geometric SE) of blood cadmium, T/S ratio, and Horvath DNAmTL.

	N	Blood Cadmium (μg/L)	N	T/S Ratio	N	Horvath DNAmTL (Kilobases)
Total	8716	0.441 (1.007)	6891	0.998 (1.003)	2126	6.568 (1.001)
Sex						
Men	4167	0.439 (1.010)	3338	0.978 (1.004)**^‡^**	1082	6.512 (1.001) **^‡^**
Women	4549	0.443 (1.009)	3553	1.017 (1.004)**^‡^**	1044	6.626 (1.001) **^‡^**
Age (in years)						
18–39	3796	0.381 (1.010) **^‡^**	2563	1.115 (1.004) **^‡^**		
40–59	2410	0.481 (1.014) **^‡^**	2113	1.005 (1.005) **^‡^**	604	6.790 (1.001) **^‡^**
≥60	2510	0.507 (1.011) **^‡^**	2215	0.872 (1.005) **^‡^**	1522	6.482 (1.001) **^‡^**
Ethnicity						
Mexican-American	2222	0.419 (1.012) **^‡^**	1656	0.977 (1.006) **^‡^**	604	6.541 (1.002) **^‡^**
Other Hispanic	443	0.411 (1.029) **^‡^**	353	1.032 (1.013) **^‡^**	131	6.610 (1.004) **^‡^**
Non-Hispanic white	4124	0.449 (1.010) **^‡^**	3525	0.987 (1.004) **^‡^**	882	6.500 (1.002) **^‡^**
Non-Hispanic black	1630	0.444 (1.017) **^‡^**	1147	1.050 (1.008) **^‡^**	439	6.731 (1.002) **^‡^**
Other ethnicity	297	0.529 (1.036) **^‡^**	210	1.021 (1.016) **^‡^**	70	6.569 (1.004) **^‡^**
Family poverty income ratio						
<1	1739	0.498 (1.017) **^‡^**	1217	1.011 (1.007) **^‡^**	360	6.557 (1.002) **^‡^**
1–3	3648	0.455 (1.011) **^‡^**	2876	0.980 (1.004) **^‡^**	958	6.526 (1.002) **^‡^**
>3	3330	0.400 (1.010) **^‡^**	2798	1.011 (1.005) **^‡^**	808	6.622 (1.002) **^‡^**
Body mass index (kg/m^2^)						
<25	3025	0.461 (1.013) **^‡^**	2167	1.022 (1.006) **^‡^**	563	6.518 (1.002) **^‡^**
25–30	3047	0.437 (1.011) **^‡^**	2524	0.989 (1.005) **^‡^**	841	6.561 (1.002) **^‡^**
>30	2644	0.423 (1.012) **^‡^**	2200	0.986 (1.005) **^‡^**	722	6.615 (1.002) **^‡^**
Smoking status						
Non-smoker	4469	0.361 (1.007) **^‡^**	3633	0.979 (1.004) **^‡^**	1273	6.563 (1.001)
ETS	1794	0.348 (1.011) **^‡^**	1370	1.011 (1.007) **^‡^**	429	6.580 (1.002)
Active smoker	2453	0.753 (1.014) **^‡^**	1888	1.026 (1.006) **^‡^**	424	6.569 (1.002)
Alcohol consumption (drinks/year)						
<12	3666	0.404 (1.010) **^‡^**	2386	0.992 (1.005)	826	6.576 (1.002)
≥12	5050	0.469 (1.009) **^‡^**	4505	1.001 (1.004)	1300	6.563 (1.001)
Hypertension						
Yes	2772	0.485 (1.011) **^‡^**	2398	0.932(1.005) **^‡^**	1286	6.543 (1.001) **^‡^**
No	5944	0.422 (1.009) **^‡^**	4493	1.036 (1.004) **^‡^**	840	6.605 (1.002) **^‡^**
Diabetes Mellitus						
Yes	977	0.449 (1.019)	829	0.921 (1.008) **^‡^**	504	6.546 (1.002)
No	7739	0.440 (1.007)	6062	1.009 (1.003) **^‡^**	1622	6.574 (1.001)
Chronic kidney disease						
Yes	551	0.529 (1.025) **^‡^**	498	0.861 (1.011) **^‡^**	283	6.421 (1.003) **^‡^**
No	8165	0.435 (1.007) **^‡^**	6393	1.010 (1.003) **^‡^**	1843	6.590 (1.001) **^‡^**
Hypercholesterolemia						
Yes	2080	0.461 (1.014) **^‡^**	1816	0.957 (1.006) **^‡^**	751	6.569 (1.002)
No	6636	0.435 (1.008) **^‡^**	5075	1.013 (1.004) **^‡^**	1375	6.567 (1.001)
History of CVD						
Yes	777	0.536 (1.022) **^‡^**	674	0.875 (1.009) **^‡^**	403	6.462 (1.002) **^‡^**
No	7939	0.432 (1.007) **^‡^**	6217	1.013 (1.003) **^‡^**	1723	6.593 (1.001) **^‡^**
History of cancer						
Yes	616	0.516 (1.023) **^‡^**	551	0.902 (1.011) **^‡^**	284	6.445 (1.003) **^‡^**
No	8100	0.436 (1.007) **^‡^**	6340	1.007 (1.003) **^‡^**	1842	6.587 (1.001) **^‡^**

**^‡^**, *p* < 0.001 Tested by two-tailed Student’s *t*-tests and one-way analysis of variance. Abbreviations: CVD: Cardiovascular disease; Horvath DNAmTL: Horvath DNA methylation predicted telomere length; ETS: Environmental tobacco smoker; T/S ratio: Telomere length relative to standard reference DNA.

**Table 2:** Adjusted regression coefficients (95% CI) for differences in ln-T/S ratio and ln-Horvath DNAmTL relative to a one-unit increase in ln-blood cadmium and ln-Horvath DNAmTL, with results weighted for sampling strategy.

	Ln-blood Cadmium (μg/L)			Ln-Horvath DNAmTL (Kilobases)		
	Unweighted No./Population Size	Adjusted β (95% CI)	*p* Value	Unweighted No./Population Size	Adjusted β (95% CI)	*p* Value
Ln-T/S ratio	6891/150,739,685			2126/65,502,614		
Model 1		−0.043 (−0.059, −0.027)	<0.001		1.782 (1.462, 2.102)	<0.001
Model 2		−0.043 (−0.059, −0.027)	<0.001		1.784 (1.464, 2.104)	<0.001
Model 3		−0.043 (−0.059, −0.027)	<0.001		1.785 (1.465, 2.105)	<0.001
Ln-Horvath DNAmTL (kilobases)	2126/32,034,886					
Model 1		−0.010 (−0.014, −0.006)	<0.001			
Model 2		−0.010 (−0.014, −0.006)	<0.001			
Model 3		−0.010 (−0.014, −0.006)	<0.001			

Model 1 adjusted for age, sex, ethnicity, family poverty income ratio, smoking, drinking, and BMI. Model 2 adjusted for model 1 plus hypertension, hypercholesterolemia, diabetes mellitus, and chronic kidney disease. Model 3 adjusted for model 2 plus a history of CVD and a history of cancer. Abbreviations: Horvath DNAmTL: Horvath DNA methylation predicted telomere length; T/S ratio: Telomere length relative to standard reference DNA.

**Table 3:** Linear regression coefficients (95% CI) of ln-T/S ratio and ln-Horvath DNAmTL per unit increase in ln-blood cadmium in subpopulation, with results weighted for sampling strategy.

	Ln-T/S Ratio			Ln-Horvath DNAmTL (Kilobases)		
	Adjusted *β* (95% CI)	*p* Value	*p* or Interaction	Adjusted *β* (95% CI)	*p* Value	*p* for Interaction
Gender			0.102			0.744
Men	−0.046 (−0.064, −0.028)	<0.001		−0.008 (−0.014, −0.002)	0.005	
Women	−0.037 (−0.059, −0.015)	0.002		−0.012 (−0.018, −0.006)	<0.001	
Age, years			0.035			0.525
18–59	−0.043 (−0.061, −0.025)	<0.001		−0.007 (−0.013, −0.001)	0.017	
≥60	−0.047 (−0.073, −0.021)	0.001		−0.012 (−0.016, −0.008)	<0.001	
Ethnicity			0.591			0.058
Non-Hispanic white	−0.045 (−0.065, −0.025)	<0.001		−0.009 (−0.015, −0.003)	0.001	
Other	−0.029 (−0.045, −0.013)	0.001		−0.010 (−0.016, −0.004)	0.001	
BMI (kg/m^2^)			0.629			0.158
<30	−0.038 (−0.058, −0.018)	0.001		−0.011 (−0.017, −0.005)	<0.001	
≥30	−0.051 (−0.073, −0.029)	<0.001		−0.007 (−0.013, −0.001)	0.038	
Smoking status			0.074			0.142
Active smoker and ETS	−0.078 (−0.110, −0.046)	<0.001		−0.010 (−0.018, −0.002)	0.024	
Non-smoker	−0.050 (−0.082, −0.018)	0.003		−0.006 (−0.010, −0.002)	0.014	
Alcohol consumption (drinks/year)			0.067			0.891
<12	−0.049 (−0.081, −0.017)	0.004		−0.010 (−0.016, −0.004)	0.002	
≥12	−0.041 (−0.057, −0.025)	<0.001		−0.009 (−0.013, −0.005)	0.001	

Adjusted for model 3. Abbreviations: BMI: Body mass index.

**Table 4:** HRs (95% CI) for all-cause, cardiovascular, and cancer mortality associated with a unit increase in ln-blood cadmium, ln-T/S ratio, and ln- Horvath DNAmTL. Results are derived from a weighted Cox regression model accounting for complex sampling design.

	**HR (95% CI)**	***p* Value**
All-cause mortality		
Ln-blood cadmium (μg/L)	1.449 (1.274–1.649)	<0.001
Ln-T/S ratio	0.675 (0.510–0.892)	0.007
Ln-Horvath DNAmTL (kilobases)	0.002 (0.001–0.028)	<0.001
Cardiovascular mortality *		
Ln-blood cadmium (μg/L)	1.385 (1.130–1.698)	0.003
Ln-T/S ratio	0.582 (0.354–0.958)	0.034
Ln-Horvath DNAmTL (kilobases)	0.007 (2.702 × 10^−5^–2.080)	0.086
Cancer-related mortality		
Ln-blood cadmium (μg/L)	1.633 (1.341–1.989)	<0.001
Ln-T/S ratio	0.589 (0.314–1.108)	0.097
Ln-Horvath DNAmTL (kilobases)	0.001 (2.264 × 10^−6^–0.112)	0.008

Adjusted for model 3 * Cardiovascular mortality: Death from heart or cerebrovascular disease Abbreviations: Horvath DNAmTL: Horvath DNA methylation predicted telomere length; HR: Hazard ratios; T/S ratio: Telomere length relative to standard reference DNA.

**Table 5:** HRs (95% CI) for all-cause mortality, cardiovascular mortality, and cancer-related mortality associated with a unit increase in ln-blood cadmium across different subgroups defined by T/S ratio and Horvath DNAmTL, derived from a weighted Cox regression model accounting for complex sampling design.

	Unweighted No./Population Size	HR	95% CI	*p* Value	*p* for Interaction
All-cause mortality					
Total	6819/149,403,806	1.445	1.251–1.670	0.001	0.255
T/S ratio < 50%ile	3390/68,074,798	1.468	1.241–1.736	<0.001	
T/S ratio ≥ 50%ile	3429/81,329,008	1.413	1.163–1.716	0.001	
Total	2031/31,051,368	1.313	1.135–1.519	0.001	0.493
Horvath DNAmTL < 50%ile	1004/13,570,298	1.355	1.056–1.738	0.019	
Horvath DNAmTL ≥ 50%ile	1027/17,481,070	1.307	1.048–1.631	0.019	
Cardiovascular mortality *					
Total	6819/149,403,806	1.407	1.106–1.790	0.007	0.508
T/S ratio < 50%ile	3390/68,074,798	1.683	1.052–2.693	0.031	
T/S ratio ≥ 50%ile	3429/81,329,008	1.296	1.018–1.650	0.036	
Total	2031/31,051,368	1.213	0.950–1.548	0.117	0.605
Horvath DNAmTL < 50%ile	1004/13,570,298	1.175	0.908–1.520	0.210	
Horvath DNAmTL ≥ 50%ile	1027/17,481,070	1.257	0.808–1.958	0.298	
Cancer-related mortality					
Total	6819/149,403,806	1.643	1.314–2.054	<0.001	0.157
T/S ratio < 50%ile	3390/68,074,798	1.751	1.267–2.422	0.001	
T/S ratio ≥ 50%ile	3429/81,329,008	1.422	1.129–1.791	0.004	
Total	2031/31,051,368	1.378	0.928–2.047	0.108	0.477
Horvath DNAmTL < 50%ile	1004/13,570,298	1.241	0.741–2.079	0.399	
Horvath DNAmTL ≥ 50%ile	1027/17,481,070	1.412	0.836–2.386	0.189	

Adjusted for Model 3. Abbreviation: Horvath DNAmTL: Horvath DNA methylation predicted telomere length; T/S ratio: HR: Hazard ratios; Telomere length relative to standard reference DNA. * Cardiovascular mortality: Death from heart or cerebrovascular disease.

## Data Availability

The datasets analyzed in this study can be accessed on the NHANES website (https://wwwn.cdc.gov/nchs/nhanes/default.aspx) (accessed on 14 September 2025).

## Supplementary Materials

The following supporting information can be downloaded at: https://www.mdpi.com/article/10.3390/life15091467/s1, Table S1: OR (95% confidence interval [CI]) of chronic diseases with one unit increase in ln-cadmium, ln-T/S ratio, and ln-Horvath DNAmTL in complex samples of logistic regression analysis, with results weighted for sampling strategy; Table S2: HR (95% CI) for all-cause, cardiovascular, and cancer mortality associated with a unit increase in ln-blood cadmium, ln-T/S ratio, and ln-Horvath DNAmTL. Results are derived from a weighted Cox regression model accounting for complex sampling design, excluding subjects with CVD and cancer; Table S3: HR (95% CI) for all-cause, cardiovascular, and cancer mortality associated with a unit increase in ln-blood cadmium, ln-T/S ratio, and ln- Horvath DNAmTL in non-smokers. Results are derived from a weighted Cox regression model accounting for complex sampling design.
