# Supplementary material for: Examining the Relationships Between Blood Cadmium, DNA Methylation Biomarker, Telomere Length, and Their Associations with Mortality in U.S. Adults
Source: Life (Basel). 2025 Sep 18;15(9):1467. doi: 10.3390/life15091467 (PMC12471579; doi:10.3390/life15091467)
Supplement: Supplementary file 1 [file life-15-01467-s001.zip › Supplemental section.pdf]

Supplementary material

Supplemental Table S1. OR (95% confidence interval [CI]) of chronic diseases with one unit increase in ln-cadmium, ln-T/S ratio, and ln-Horvath DNAmTL in complex samples of logistic regression analysis, with results weighted for sampling strategy.

	Ln-blood cadmium (ug/L)		Ln-T/S ratio		Ln-Horvath DNAmTL (base pairs))	
	OR (95% CI)	P value	OR (95% CI)	P value	OR (95% CI)	P value
Hypertension	0.992 (0.857—1.148)	0.914	1.458 (1.073—1.981)	0.018	0.147 (0.002—10.558)	0.367
Diabetes Mellitus	0.693 (0.541—0.887)	0.005	1.097 (0.620—1.941)	0.743	0.023 (0.000—1.213)	0.061
Chronic kidney disease	1.401 (1.048—1.872)	0.024	1.045 (0.558—1.958)	0.886	0.05 (0.000—6.828)	0.223
Hypercholesterolemia	1.096 (0.957—1.255)	0.177	0.824 (0.567—1.197)	0.297	1.723 (0.035—5.603)	0.515
History of CVD	1.291 (1.039—1.604)	0.023	0.524 (0.282—0.972)	0.041	0.011 (0.000—0.407)	0.016
History of cancer	1.341 (1.076—1,671)	0.011	1.041 (0.604—1.796)	0.880	0.055 (0.000—6.106)	0.218

Adjusted for Model 1: Age, sex, ethnicity, family poverty income ratio, smoking, drinking, and BMI.

Abbreviations: Horvath DNAmTL: Horvath DNA methylation predicted telomere length; Odds ratios: OR; T/S ratio: Telomere length relative to standard reference DNA.

Supplemental Table S2. HR (95% CI) for all-cause, cardiovascular, and cancer mortality associated with a unit increase in ln-blood cadmium, ln-T/S ratio, and ln-Horvath DNAmTL. Results are derived from a weighted Cox regression model accounting for complex sampling design, excluding subjects with CVD and cancer.

	Separate analysis	
	HR (95% CI)	*P* value
All-cause mortality		
Ln-blood cadmium (ug/L)	1.425 (1.215—1.672)	<0.001
Ln-T/S ratio	0.610 (0.419—0.887)	0.011
Ln-Horvath DNAmTL (base pairs)	0.007 (0.001—0.133)	0.002
Cardiovascular mortality*		
Ln-blood cadmium (ug/L)	1.410 (1.068—1.860)	0.017
Ln-T/S ratio	0.704 (0.389—1.273)	0.235
Ln-Horvath DNAmTL (base pairs)	0.016 (8.797×10^{-6}—30.524)	0.273
Cancer-related mortality		
Ln-blood cadmium (ug/L)	1.435 (1.129—1.824)	0.005
Ln-T/S ratio	0.521 (0.247—1.100)	0.085
Ln-Horvath DNAmTL (base pairs)	0.001 (1.414×10^{-6}—0.099)	0.007

Adjusted for model 3

*Cardiovascular mortality: Death from heart or cerebrovascular disease

Abbreviations: Horvath DNAmTL: Horvath DNA methylation predicted telomere length; T/S ratio: HR: Hazard ratios; Telomere length relative to standard reference

DNA.

Supplemental Table S3. HR (95% CI) for all-cause, cardiovascular, and cancer mortality associated with a unit increase in ln-blood cadmium, ln-T/S ratio, and ln-Horvath DNAmTL in non-smokers. Results are derived from a weighted Cox regression model accounting for complex sampling design

	HR (95% CI)	*P* value
All-cause mortality		
Ln-blood cadmium (ug/L)	1.205 (1.065—1.364)	0.004
Ln-T/S ratio	0.643 (0.465—0.890)	0.009
Ln-Horvath DNAmTL (base pairs)	0.003 (0.000—0.044)	<0.001
Cardiovascular mortality*		
Ln-blood cadmium (ug/L)	1.328 (1.067—1.653)	0.013
Ln-T/S ratio	0.635 (0.378—1.067)	0.084
Ln-Horvath DNAmTL (base pairs)	0.013 (2.653×10^{-5}—6.535)	0.164
Cancer-related mortality		
Ln-blood cadmium (ug/L)	1.454 (1.040—2.032)	0.030
Ln-T/S ratio	0.332 (0.156—0.706)	0.006
Ln-Horvath DNAmTL (base pairs)	0.001 (1.631×10^{-6}—0.078)	0.008

Adjusted for model 3

*Cardiovascular mortality: Death from heart or cerebrovascular disease

Abbreviations: Horvath DNAmTL: Horvath DNA methylation predicted telomere length; HR: Hazard ratios; T/S ratio: Telomere length relative to standard reference DNA,
